# Predicting fitness to practise events in international medical graduates who registered as UK doctors via the Professional and Linguistic Assessments Board (PLAB) system: a national cohort study

**DOI:** 10.1186/s12916-017-0829-1

**Published:** 2017-03-20

**Authors:** Paul A. Tiffin, Lewis W. Paton, Lazaro M. Mwandigha, John C. McLachlan, Jan Illing

**Affiliations:** 1 Department of Health Sciences, University of York, Heslington, York, YO10 5DD UK; 2 School for Medicine, Pharmacy and Health, Durham University Queen’s Campus, Thornaby, TS17 6BH UK; 3 School of Medical Education, The Medical School, Newcastle University|, Newcastle-upon-Tyne, NE1 7RU UK

**Keywords:** International medical graduates, Professionalism, Medical regulation, Fitness to practise

## Abstract

**Background:**

International medical graduates working in the UK are more likely to be censured in relation to fitness to practise compared to home graduates. Performance on the General Medical Council’s (GMC’s) Professional and Linguistic Assessments Board (PLAB) tests and English fluency have previously been shown to predict later educational performance in this group of doctors. It is unknown whether the PLAB system is also a valid predictor of unprofessional behaviour and malpractice. The findings would have implications for regulatory policy.

**Methods:**

This was an observational study linking data relating to fitness to practise events (referral or censure), PLAB performance, demographic variables and English language competence, as evaluated via the International English Language Test System (IELTS). Data from 27,330 international medical graduates registered with the GMC were analysed, including 210 doctors who had been sanctioned in relation to at least one fitness to practise issue. The main outcome was risk of eventual censure (including a warning).

**Results:**

The significant univariable educational predictors of eventual censure (versus no censures or referrals) were lower PLAB part 1 (hazard ratio [HR], 0.99; 95% confidence interval, 0.98 to 1.00) and part 2 scores (HR, 0.94; 0.91 to 0.97) at first sitting, multiple attempts at both parts of the PLAB, lower IELTS reading (HR, 0.79; 0.65 to 0.94) and listening scores (HR, 0.76; 0.62 to 0.93) and higher IELTS speaking scores (HR, 1.28; 1.04 to 1.57). Multiple resits at either part of the PLAB and higher IELTS speaking score (HR, 1.49; 1.20 to 1.84) were also independent predictors of censure. We estimated that the proposed limit of four attempts at both parts of the PLAB would reduce the risk in this entire group by only approximately two censures per 5 years in this group of doctors.

**Conclusions:**

Making the PLAB, or any replacement assessment, more stringent and raising the required standards of English reading and listening may result in fewer fitness to practice events in international medical graduates. However, the number of PLAB resits permitted would have to be further capped to meaningfully impact the risk of sanctions in this group of doctors.

**Electronic supplementary material:**

The online version of this article (doi:10.1186/s12916-017-0829-1) contains supplementary material, which is available to authorized users.

## Background

The healthcare workforce is internationalised and globalised [1]. In particular, the health services of developed countries rely heavily on medical graduates who qualified elsewhere, especially in less popular specialities such as psychiatry [2]. In the UK, in 2016, 26% of the doctors registered with the regulatory body, the General Medical Council (GMC), qualified from outside the European Economic Area (EEA) [3]. There are some suggestions in the UK that, with changes in immigration regulations and European employment law, the proportion of European-trained doctors has increased [2]. Subsequent to the likely departure of the UK from the EEA, non-British European doctors may also be required to sit tests before registration, though such issues are yet to be clarified. For the purposes of this report we define international medical graduates as those who qualified from a country outside of the EEA.

For a doctor to be legally allowed to practise medicine in the UK they must fulfil the requirements of the 1983 Medical Act [4]. For international medical graduates this mainly involves passing both parts of the Professional and Linguistic Assessments Board (PLAB) test, though other routes to registration are available, especially for more experienced practitioners. The first part of the PLAB evaluates the medical knowledge of candidates, as relevant to the UK. It is a 3 hour exam with 200 multiple choice questions where the candidate must select the single best answer. The test covers the following domains: ‘applying knowledge and experience to practice’, ‘clinical care’, ‘assessment’, and ‘clinical management’. The pass mark is decided by a modified version of the Angoff method, whereby experts decide the minimum scores that would be acceptable for the test items [5]. Part 2 of the PLAB is a practical evaluation of clinical skills. At the time of the study it consisted of 14 objective structured clinical examination stations. Each station consisted of a 5 minute clinical scenario where candidates were observed by a lone examiner and rated on their performance. The skills assessed were ‘clinical examination’, ‘practical skills’, ‘communication skills’, and ‘history taking’. It should be noted that, since this study was conducted, changes have been made to the format of part 2 of the PLAB, including an increase in the number and length of scenarios [6]. The pass mark for part 2 was decided via the borderline group scoring method, which involved weighting the scores for the stations [7]. The weightings themselves were decided according to expert opinion of the score expected of a ‘minimally competent’ candidate. The weightings were also informed by the scores obtained by previous cohorts. Part 2 of the test had to be passed within 3 years of passing part 1. Up until September 2017, an unlimited number of attempts at parts 1 and 2 of the PLAB are permitted. Subsequently, the number of attempts at each part will be limited to four.

In order to be eligible to sit the PLAB test doctors must have an acceptable medical degree from a recognised institution [8], have had at least 12 months of postgraduate clinical experience and produce evidence of competency in the English language. The latter is normally provided by having passed an International English Language System (IELTS) assessment to an appropriate level [9]. The IELTS test is in four parts, namely ‘listening’, ‘speaking’, ‘reading’, and ‘writing’. For the listening section, candidates must listen to a series of speech samples, which include both social scenarios as well as those related to education and training, before answering comprehension questions. For the speaking subtest a face-to-face interview is conducted which involves conversation as well as being prompted to provide information or opinions on specific themes. The reading section consists of a comprehension test based on passages of a text taken from journals, newspapers, etc. Whilst the texts are often quite academic in nature, they are selected for general interest. For the writing subtest, candidates are presented with facts, figures or visually presented material, such as graphs, and must describe them in words. In addition, for the writing section, test-takers must also write about a topic they are given. Thus, although overall competence in the use of English is assessed, the IELTS has a somewhat academic flavour to it. This is in keeping with one of the main roles of the test, which is to ensure language fluency in those who wish to train or study in English speaking countries. Each part of the IELTS is graded between band one (non-user) and band nine (expert user). The test can be taken as many times as required to obtain the desired score. Up until June 2014, in order to be eligible to sit the PLAB test, an overall IELTS score of at least 7.0 was required. Subsequently, the standard of language fluency required has been raised and an overall score of 7.5 is now required [10]. Once all these requirements are met, the GMC also considers an applicant’s fitness to practise (FtP) before the final decision is made about whether the doctor’s name is placed on the GMC’s list of registered medical practitioners.

Conceptually, the PLAB test and IELTS could be viewed as measuring underlying constructs that are likely to be linked to the risk of future FtP issues. The PLAB part 1 is a test of semantic medical knowledge. PLAB part 2 is designed to evaluate the extent to which this medical knowledge can be applied in context, and also rates procedural and technical skills, such as the examination of patients. If these aspects of semantic or procedural knowledge are deficient, then the risk of clinically related FtP issues would increase. However, the vast majority of FtP concerns that result in actual censure are related to personal conduct rather than primarily clinical competence (see later). Nevertheless, PLAB part 2 could be considered as also tapping into ‘softer’ skills that relate to interpersonal functioning and could be associated with the probability of future FtP events occurring. For example, the ‘communication’ section of PLAB part 2 should have captured the extent to which a candidate displays professional behaviours and effective interactions with a role-playing patient in an objective structured clinical examination situation. Likewise, the IELTS scores are likely to reflect a certain degree of cultural, as well as linguistic, competence in candidates; language and culture are difficult to separate. It is also possible that both language- and culture-based misunderstandings between individuals may lead to complaints, and FtP referrals, even if eventually there is found to be no cause for concern. Moreover, both IELTS and the PLAB test will evaluate more generic, second order attributes, such as general cognitive ability and conscientiousness, which are necessary to achieve relatively high scores. These, too, are likely to be associated with the risk of FtP issues.

Despite a continued reliance in Western countries on doctors trained elsewhere, there has been a concern regarding whether those practitioners who obtained their primary medical degree from outwith the host nation can be considered equivalent to home graduates. Specifically, issues relating to preparedness for practice in particular with regard to communication, cultural competence, clinical knowledge and skills could impact on the ability to deliver care that is comparable in safety and quality to doctors graduating from the host country. Previously, two parallel studies observed that, compared to UK graduates, international medical graduates performed, on average, more poorly on evaluations of postgraduate educational performance [11, 12]. However, in both cases, these differences diminished in magnitude for those international doctors who demonstrated higher performance on the IELTS and the PLAB test. It is unknown whether such educational discrepancies between native and international graduates translate into poorer clinical outcomes for patients. However, one North American study observed poorer outcomes in the cardiology patients of doctors who were US citizens but had graduated abroad (in contrast to US graduates and non-US international medical graduates) [13]. This is despite all practising US doctors being required to sit the same national licensing exam [14].

The GMC has a statutory duty to investigate and, where appropriate, take action against doctors when they receive information that raises doubts regarding their FtP medicine. Such FtP processes are triggered by complaints or expressions of concern that can come from any source, including members of the public, colleagues, employers or the police [15]. Such enquiries are initially triaged and a decision on further action is made according to whether the threshold for further investigation is met [16]. Concerns that are unlikely to imply that the doctor’s fitness is impaired are either closed at this triage stage or referred to the practitioner’s employer, who may investigate them locally. Where the concerns are more serious in nature then an investigation may follow; this may involve obtaining further information from reports or witnesses and assessments of the doctor’s health. Once the investigations are concluded the allocated medical and non-medical case examiners reassess the case in order to decide whether further action is appropriate. Most result in no further action at this stage. In cases considered less serious, but where there is clear evidence that fitness was impaired, doctors may be offered the opportunity to receive a written warning and/or agree to specific undertakings in their practice (for example, agreeing not to perform a specific surgical procedure) [17]. In a small number of cases deemed more serious, or where doctors refuse to accept a warning and/or agree to undertakings, the case may be referred to the Medical Practitioners Tribunal Service [18]. This tribunal service is subservient to a court, is independent of the GMC and is composed of a panel of medical and legal experts as well as lay members. The tribunal has the power to sanction doctors who are found to be unfit to practise, including the ability to suspend or erase a doctor from the registered list of medical practitioners.

It is well established that non-UK medical graduates are over-represented in FtP referrals, though those graduating from the EEA, and who are not required to sit the PLAB, are at the highest risk of eventual censure [2]. Indeed, doctors who qualified outside of the UK are more likely to receive ‘high impact’ decisions at every stage of the FtP process [19]. Thus, they are much more likely than UK medical graduates to be suspended or erased from the medical register held by the GMC. Other demographic factors, such as sex, are also associated with the risk of FtP issues occurring; a meta-analysis reported that, on average, male doctors had nearly two and a half times the odds of being subject to medico-legal action, compared to female doctors [20].

In parallel to issues regarding the equivalence of UK and international graduates, concerns have emerged regarding discrimination and fairness towards both non-UK doctors and those who trained in the UK and are from Black and Minority Ethnic groups [21]. In particular, one study highlighted that differences in pass rates in the Clinical Skills Assessment component of the Membership of the Royal College of General Practitioners exam between White and Black and Minority Ethnic candidates persisted even after controlling for the influences of potential confounding variables, including performance at the knowledge component of the test [22]. These concerns were debated in the High Court when the British Association of Physicians of Indian Origin took (ultimately unsuccessful) action against the GMC and the Royal College of General Practitioners [23]. Whilst racial discrimination has not been comprehensively ruled out, the findings from a detailed linguistic study of candidates undertaking the Clinical Skills Assessment suggest more subtle factors relating to culture and communication may be driving the differential attainment rates [24].

The present study was conducted as part of a programme of research to explore the validity of the PLAB test [25]. In addition, the linking of large scale data related to FtP, English language competence and demographics to the PLAB test scores allowed us to seek further evidence that could shed light on the underlying reasons for observed differences in professional performance between UK and international medical graduates. Our aim was to evaluate the validity of the PLAB system (of which the IELTS could be considered a component) with respect to whether the scores demonstrated an ability to predict the risk of subsequent FtP issues in international medical graduates registering via this route. The study was also an opportunity to evaluate the extent to which the proposed restrictions placed on the number of times the PLAB test could be taken might be expected to impact on the future rate of FtP events in this group of doctors working in the UK. The findings also have significant international implications, particularly with regard to re-sits. For instance, the Australian Medical Council currently does not restrict the number of re-sits candidates undertake in professional exams, because their legal guidance has been that there is insufficient evidence to justify such a restriction. This paper provides such evidence.

## Methods

### Data

For these analyses, PLAB performance data were available on a total of 30,049 candidates. Of these, 29,166 were recorded as having obtained their primary medical qualification from outwith the EEA. Of this group, 27,399 candidates were recorded as having passed part 2 of the PLAB in the study timeframe (see below). A further five candidates were excluded from the analyses as they were recorded as never having passed part 1 of the PLAB. In three of these apparently anomalous cases, PLAB part 1 was listed as having been taken after this period, but paradoxically after part 2 had been recorded as passed. These were assumed to be data entry errors. Further investigation by the GMC indicated that this observation was likely to be due to how the dataset was constructed for transfer for the present study rather than reflecting the actual registration history of the doctors concerned. Consequently, as a precautionary measure, data relating to these individuals were excluded from the final dataset for analysis. A further eight candidates had a record of PLAB part 1 having been passed, but again, after part 2 was reported as passed. Again, these were assumed to be data entry errors but the observations were excluded from the final analyses as a precaution. This left data on 27,386 international medical graduates who had registered via the PLAB system. All these doctors had taken the PLAB part 1 sitting at which they had passed between July 4, 2000, and September 8, 2011. They had taken the PLAB part 2 exam, which they had passed between June 13, 2001, and December 7, 2011. For these analyses, PLAB performance, when treated as a continuous variable, was taken as the score achieved, relative to the pass mark at that sitting, at first attempt.

These scores were combined with socio-demographic data from the List of Registered Medical Practitioners with date of birth, sex, and date of first registration with the GMC. Ethnicity, which is provided to the GMC on a voluntary basis, was only recorded in a minority of the final sample cases (33%) and, due to this degree of absence, it was excluded from the analyses as a predictor. To ensure the data were anonymous when shared with the researchers, the GMC restricted the date variables to years (i.e. *YearOfBirth*, *YearOfProvReg*, *YearOfFirstReg*). In 56 cases, the data provided by the GMC suggested year of registration preceded the year that PLAB part 2 was passed. Further investigation by the GMC identified that historical changes to registration rules explained these anomalies. Again, data relating to these doctors were removed from the final dataset for analysis as a precaution. This left a total of 27,330 international PLAB medical graduates in the dataset.

The overall IELTS scores, graded for these doctors, ranged from 7.0 to 9.0 (with 7.0 being the previously lowest acceptable score for registration and 9.0 representing the highest achievable grade). The IELTS scores were potentially available for 25,768 of the final set of international medical PLAB graduates. It was noted that the speaking subtest score was missing in two cases. Additionally, in eight cases, the overall IELTS scores were less than 7.0. The IELTS scores from these latter eight cases were recoded as missing as a precaution (the doctors may have provided alternative evidence of English fluency in these instances). The median overall IELTS score for the cohort was 7.5 (inter-quartile range 7.0 to 7.5). This would be categorised as somewhere intermediate between a ‘good user’ and a ‘very good user’ of English, i.e. someone with a generally fully operational command of the language with only infrequent inaccuracies or misunderstandings [9].

Data were also available on FtP procedures on cases closed by the GMC between the start of 2006 and the end of 2012, though this did not include any cases referred after the end of 2011. These were linked to the PLAB performance and list of registered medical practitioners’ data via a unique identifier based on the GMC registration number. After the exclusions mentioned above, data on FtP processes were available for 1319 cases relating to 1182 international medical graduates who registered via the PLAB system, with 215 of these (18.2%) eventually receiving some form of censure.

There were nine categories of FtP allegation (Table 2). The nature of the allegation was categorised by the GMC and the researchers did not have access to the text descriptions of the concerns or allegations relating to the referrals. Many cases that were closed during the study period (2006 to 2011, inclusive) involved more than one type of allegation, with a total of 1607 separate allegation domains relating to the 1319 cases. However, separate allegations relating to the same case are likely to be associated in some way (for example, they could have been made by a single complainant or complainants who were known to, or related to, each other). Therefore, for the purposes of analysis, a doctor was indicated as having an FtP concern relating to a particular category if there was at least one allegation reported in relation to that aspect of practise or conduct.

Table 2 is informative as it shows that the ‘conversion rates’ for cases in relation to some categories of allegation are much higher than others.^1^ For example, whilst only around 12% of doctors with an allegation against them relating to the standard of clinical care received some kind of censure, the majority (around 70%) who had allegations related to a GMC compliance order had some action taken against them.^2^ The largest number of allegations that result in sanction for both international (*n* = 162) and UK medical graduates (*n* = 271) were in relation to probity.

The main aim of the study was to explore the predictors of actual censure, rather than merely referral in relation to FtP concerns. In this case, censure, as the primary outcome, was defined as any FtP referral that eventually resulted in a sanction of any kind, including a warning being issued. We also sub-categorised censures into those that were received purely in relation to ‘non-clinical concerns’ (as opposed to censures which involved clinical issues, with or without ‘non-clinical concerns’). Non-clinical concerns included all the categories depicted in Table 2, with the obvious exception of ‘closed at triage’. While it would have been desirable to distinguish censures received for ‘purely clinical concerns’, the very small number of such cases (*n* = 12) precluded such an analysis.

Whilst not the primary aim of the study a series of analyses were conducted in order to evaluate the predictors of a referral in relation to a FtP concern, whether or not censure ensued. For this purpose, doctors were classified into three groups according to the nature of the allegation (or multiple allegations) against them, namely those who had ‘purely clinical’ issues raised (*n* = 196); those where only ‘non-clinical’ issues were reported (including health; *n* = 436), or ‘mixed’ grounds (both clinical and non-clinical allegations raised; *n* = 163). The remainder of doctors where all issues relating to FtP were raised were discounted as having been dismissed at triage (*n* = 387). Unfortunately, the numbers of doctors falling into each separate category were too small to adequately power a detailed subgroup analysis. However, it was decided to run an analysis that discriminated between those referred in relation to ‘purely clinical’ concerns and those where ‘non-clinical concerns’ were raised (with or without ‘clinical concerns’). Cases where concerns were dismissed at triage were not included as there may have been no case to answer or such issues may have been better dealt with by local employers, and it is uncertain the extent to which these concerns would have been processed in this way, or raised at all. At the time of writing an increasing proportion of such allegations appear to be dealt with locally (hence the recent fall in the number of referrals).

The relationship between the subscale scores of the two parts of the PLAB tests (e.g. ‘history taking’, etc.) and FtP events were explored via univariable analyses. However, these sub-tests are not standardised between exam sittings (hence scores could not be equated across exam cohorts). No distinct patterns were noted, though this could have been at least partly due to a lack of score standardisation. Consequently, the results in relation to the sub-test components of the PLAB are not reported.

### Survival analysis

Previously, some of these data had been analysed using logistic regression [26]. However, as the ‘exposure time’ (i.e. time from registration to end of study period) varied depending on when the doctor was first registered, it was decided to re-analyse the data using a survival analysis approach which is better adapted to dealing with this issue. In this case ‘date of registration’ was defined by the date of the sitting at which PLAB part 2 had been passed. This was because, to anonymise the data, no exact date for registration was made available to the research team, only the year, so using the PLAB passing date improved the precision of the estimate of the exposure period. Thus, we assumed the doctor started practising in the UK shortly after part 2 of the PLAB was passed (permitting registration). ‘Time to event’ was coded as time to the first FtP allegation minus the ‘entry time’. Entry time was either the date of passing part 2 of the PLAB or the start of the study observation period, whichever was later. For the purposes of this study, the observation period was defined as starting January 1, 2006, when FtP events that were closed during that period started to be logged. There were 18 FtP events that were closed during the study period but that had been initially referred prior to the start of 2006. These were excluded from the survival analyses. This left 1301 cases relating to 1168 doctors, of which 210 eventually received some form of censure. For those with IELTS scores, these numbers were slightly reduced at 1078 doctors, with at least one allegation against them and 197 (18.3%) who eventually received some form of censure. The observation period was considered to have terminated at the end of 2011, when no referrals were included, though some cases were not closed until the end of 2012. Where there were no FtP referrals, or, for sub-group analysis, no FtP referrals of that category (e.g. for ‘purely clinical concerns’) the event time was coded as the last period of observation (i.e. end of 2011, when the last case closed was referred). Thus, where no relevant event had occurred, the observation was censored. In only seven cases was a doctor referred to the GMC and no censure initially resulted, but on a second occasion a new allegation did conclude with a censure of some kind. Therefore, for the purposes of survival analyses the ‘time to event’ (an allegation relating to a case resulting in censure) was defined as the time from passing PLAB part 2 to the time a first case was logged (relating to at least one allegation) for that doctor, whether or not it resulted in action. This approach was taken as it assumed the initial concerns, though not resulting in sanction, may have actually had some basis, given the subsequent referral resulting in censure.

It should be noted that, in two cases, FtP allegations were recorded before part 2 of the PLAB had been passed. These ‘time to events’ were recoded as zero to avoid the use of negative numbers. They could have reflected concerns raised with international doctors not yet registered but on clinical attachment – in one case, an FtP concern occurred just over 2 years before PLAB part 2 had been passed; in another case, it was around 4 months previous to passing PLAB part 2; in a third case, PLAB part 2 had been passed just prior to the date of an FtP allegation but the year of registration was the following year (in this case, at least one month after the recorded allegation date).

A series of survival analyses were conducted in order to evaluate the extent to which PLAB and IELTS performance and demographic variables predicted the risk of referral for FtP and eventual censure. Initially, a series of univariable analyses were conducted. Variables that did not show at least a strong trend (*P* < 0.2) towards influencing the risk were not included in the later multivariable models. Multivariable model building proceeded in a forward stepwise way, though in our results we present the full models, including variables where the final, independent predictive ability was not significant at the *P* < 0.05 level.

Previously, a greater number of resits at PLAB part 1 or part 2 has been shown to predict poorer postgraduate educational outcomes in international medical graduates [12, 26]. Therefore, as before, we also categorised PLAB graduates based on the number of resits at both parts of PLAB in order to explore any association with later FtP events. For part 1, candidates were categorised as having one, two, three or four or more sittings before passing; for part 2, candidates as having one, two or three or more sittings before passing. These categories were chosen because few doctors had taken PLAB part 1 more than four times and PLAB part 2 more than three times. As before, during analysis, the ‘base category’ was swapped several times so that all permutations of comparison could be evaluated (e.g. pass at first attempt vs. passed at third attempt, etc.) within the regression analyses.

Survival analyses produce an estimate of the ‘hazard ratio’ (HR) for a predictor variable that is a postulated to be associated with the risk of an outcome occurring [27]. The HR represents the ratio of probabilities that the outcome event will occur with the risk (or protective) factor present as opposed to absent, over any given time period. As the risk is assumed to be constant over time (the ‘proportional hazard assumption’) the unit of time is irrelevant. For continuous variables the HR reflects the change in relative risk for every additional unit in the predictor.

In the present analyses, in order to evaluate whether the proportional hazard assumption was fulfilled, we used the ‘phtest’ command in STATA. This tests, for individual variables, whether the slope derived from a linear regression of the Schoenfeld residuals against time significantly differs from zero; in such a case there is evidence that the proportional hazard assumption is violated [28].

The data were managed and analysed using STATA 14.2 Multiprocessor (MP) version [29].

## Results

### Descriptive statistics

The demographic characteristics and PLAB test performance of the cohort are summarised in Table 1. As can be seen, the majority of doctors in the study were male (60%) and the mean age was approximately 31 years old at registration. The mean scores at first attempt were approximately six or seven points above the pass mark for both parts, highlighting that most candidates pass the tests at first sitting.

**Table 1:** Demographics and overall performance in the Professional and Linguistic Assessments Board (PLAB) test for the cohort

Characteristic	
Age at registration, mean (SD)	30.79 years (4.99)
Male sex	16,502/10,828 (60.38%)
PLAB part 1 score at first attempt, relative to pass, mean (SD)	7.47 (19.29)
PLAB part 2 score at first attempt, relative to pass, mean (SD)	6.00 (4.58)
Mean no. of sittings – PLAB part 1, mean (SD)	1.49 (1.01)
Mean no. of sittings – PLAB part 2, mean (SD)	1.24 (0.53)

The breakdown of FtP cases referred and censures according to the category of allegation are depicted in Table 2. Thus, the conversion rates (from referral to sanction) for PLAB and UK graduates for different categories of allegation can be viewed. For international medical graduates, of 360 doctors with at least one allegation against them related to clinical care, only 43 (11.9%) were eventually censured in relation to those concerns. In contrast, of 663 UK graduates with allegations relating to clinical care, only 41 were censured in relation to these issues (6.2%). Thus, few allegations against PLAB international medical graduates in relation to clinical care are found to warrant censure but even fewer are deemed to warrant censure in the case of UK graduates. The degree of correlation between the continuous predictor variables was also evaluated. The correlations were in the expected direction, though it was noted that, unlike the other language test scores, the IELTS speaking rating was positively correlated with age at registration (*r* = 0.06, *P* < 0.0001). The full results are shown in Additional file 1: Table S1.

**Table 2:** A breakdown of the number (and percentages) of Fitness to Practise referrals and eventual censures by allegation category, for both international medical Professional and Linguistic Assessments Board (PLAB) graduates and UK qualifying doctors

International Medical Graduates registering via the PLAB				UK Medical Graduates		
Allegation category	No. of allegations (% of allegations) *n* = 1607^a^	Relating to no. of doctors (% of doctors) *n* = 1182	No of doctors censured (% of doctors with allegations censured in that category) *n* = 215^b^	No. of allegations (% of allegations) *n* = 2821	Relating to no. of doctors (% of doctors) *n* = 2136	No. of doctors censured (% of doctors with allegations censured in that category) *n* = 342^a^
Closed at triage	458 (28.5)	439 (37)	NA	850 (30.1)	812 (38.1)	NA
Clinical care	368 (22.9)	360 (30.5)	43 (11.9)	681 (24.1)	663 (31.1)	41 (6.2)
Compliance	13 (0.8)	13 (1.1)	9 (69.2)	6 (0.2)	6 (0.3)	3 (50.0)
Health	56 (3.5)	55 (4.7)	33 (60.0)	191 (6.8)	185 (8.7)	113 (61.1)
Breaches of GMP^c^	46 (2.9)	46 (3.9)	18 (39.1)	40 (1.4)	37 (1.73)	8 (21.6)
Probity	418 (25.9)	389 (32.9)	162 (41.6)	625 (22.2)	593 (27.8)	271 (45.7)
Relationship with patient	147 (9.1)	145 (12.3)	14 (9.7)	319 (11.3)	307 (14.4)	16 (5.2)
Teaching and supervision	3 (0.2)	3 (0.3)	2 (66.7)	9 (0.3)	9 (0.4)	3 (33.3)
Relationship with colleagues	89 (5.5)	89 (7.5)	32 (36.0)	100 (3.5)	97 (4.5)	23 (23.7)
Other	10 (0.6)	10 (0.8)	0 (0)	3 (0.1)	3 (0.1)	0 (0)

^a^Many cases involved more than one type of allegation, with a total of 1607 separate allegation domains relating to the 1319 cases

^b^Referrals and censures can cover multiple categories therefore these values may not sum to a total

^c^GMP – Good Medical Practice, the duties of a doctor registered with the General Medical Council

### Survival analysis: univariable analyses

As the vast majority of referrals in relation to FtP concerns result in no further action, we have focussed on the findings in relation to actual censure. However, as a referral is a necessary prerequisite to receiving censure, we indicate the overall findings in this respect and make the full results available in Additional file 1.

In this sample of international medical graduates who registered with the GMC, having demonstrated their clinical knowledge and skills via the PLAB, we observed that most demographic and PLAB performance indicators were predictive of an FtP referral occurring. The only exceptions to this were the overall IELTS score as well as the IELTS writing subtest score. Some differences in the pattern of predictors were noted when the type of concerns was categorised into different types (e.g. purely clinical vs. other type). The full results are depicted in Additional file 1: Tables S2–4.

**Fig. 1:**
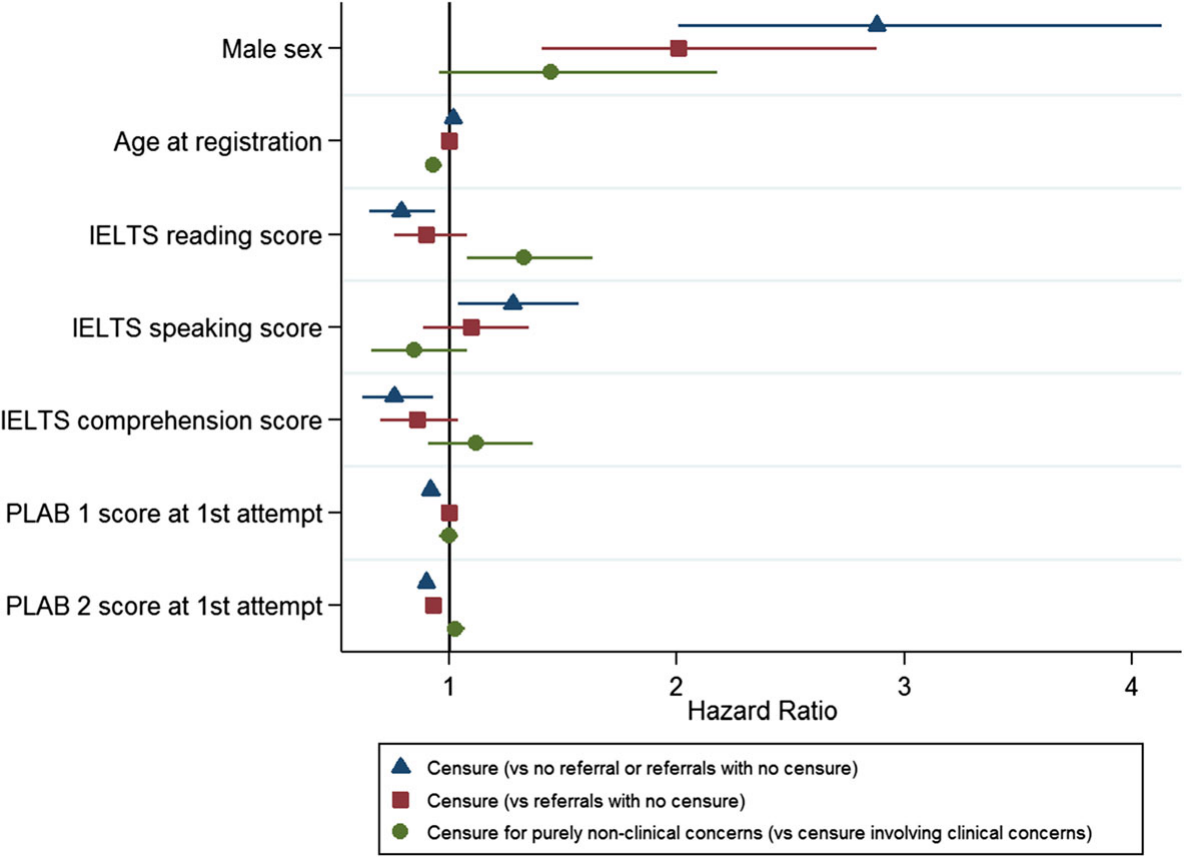
Graph showing the risk of being eventually censured for fitness to practise issues in a sample of international medical graduates in relation to seven predictors. The coefficients depicted are hazard ratios derived from univariable survival analyses, with associated 95% confidence intervals. *Blue triangles* represent the estimated risk of being censured (versus not being censured, referred or referred without eventual censure *n* = 27,330). *Red squares* represent the risk of being eventually censured only in the group referred (*n* = 1168). *Green circles* represent the risk of being censured purely in relation to non-clinical (i.e. professionalism) concerns versus censure, which involves some clinical component (with or without professionalism issues *n* = 210)

**Fig. 2:**
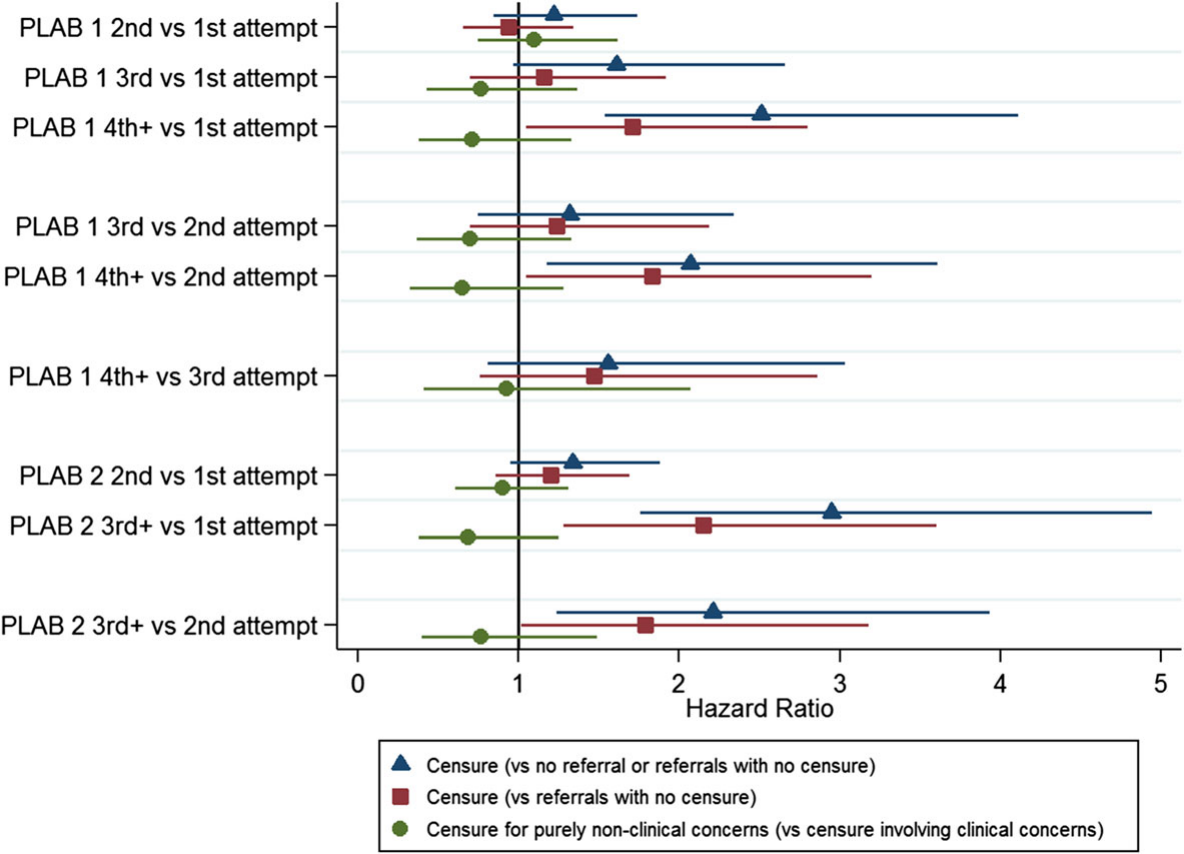
Graph showing the risk of being eventually censured for fitness to practise issues in a sample of international medical graduates in relation to the number of attempts at both parts of the Professional and Linguistic Assessments Board exam. The coefficients depicted are hazard ratios derived from univariable survival analyses, with associated 95% confidence intervals. *Blue triangles* represent the estimated risk of being censured (versus not being censured, referred or referred without eventual censure *n* = 27,330). *Red squares* represent the risk of being eventually censured only in the group referred (*n* = 1168). *Green circles* represent the risk of being censured purely in relation to non-clinical (i.e. professionalism) concerns versus censure, which involves some clinical component (with or without professionalism issues *n* = 210)

In the study cohort, 210 doctors were recorded as having been both referred and receiving censure for FtP concerns during the exposure period, as defined for the purposes of the survival analysis. The results of the survival analyses for the prediction of eventually receiving a censure are depicted in Figs. 1 and 2. Figure 1 depicts the results in relation to seven predictors, including the PLAB scores, relative to the pass mark at first attempt. Note that Fig. 1 only displays coefficients related to variables that were statistically significant (*P* < 0.05) predictors in at least one instance. Figure 2 illustrates the estimated HRs in relation to the number of attempts at both parts of the PLAB test. As can be seen from the figures (the coefficients are represented as blue triangles) significant predictors of being censured for FtP issues (compared to no censure, no referral or referral only) were male sex (HR, 2.88; 95% confidence interval, 2.01 to 4.13), higher IELTS speaking score (HR, 1.28; 1.04 to 1.57) and multiple attempts at PLAB part 1 (HR, 1.49; 1.12 to 1.97) or part 2 (HR, 1.57; 1.16 to 2.13). Higher scores at IELTS reading (HR, 0.79; 0.65 to 0.94) and listening (HR, 0.76; 0.62 to 0.93) and both parts 1 (HR, 0.99; 0.98 to 1.00) and 2 (HR, 0.94; 0.91 to 0.97) of the PLAB at first sitting were protective against the risk of eventual censure. The full results are also detailed in Additional file 1: Table S5. The HR for male sex implies that, on average, the risk of censure in relation to FtP for a male at any point is almost three times that of a female international medical graduate. When interpreting the IELTS scores one must consider that the grade bandings ascend in increments of 0.5 of a point. Thus, an HR of 0.79 for the IELTS reading score implies that, on average, the relative risk of receiving a censure decreases by roughly 20% for every additional point achieved, for example, a candidate achieving a score of 8.0 rather than 7.0 on that particular domain. When interpreting the results in relation to number of sittings of the PLAB, the comparator (base) category must be borne in mind. In this case, the base category was set to a single sitting (i.e. pass at first attempt) at each of the two parts. Therefore, we would interpret a HR of 1.49 for multiple attempts at PLAB part 1 as meaning that, on average, those taking the exam more than once would be at a roughly 50% increased risk of receiving a censure compared to those who passed the test at first sitting. When considering the coefficients associated with the PLAB performance in terms of score achieved, the metric of the variable must also be remembered. As the PLAB scores are not standardised between cohorts the values are entered as ‘score relative to pass mark for that sitting, at first attempt’. Thus, for example, an HR of 0.94 for PLAB part 2 performance indicates that, on average, the risk of censure falls by approximately 6% for every point scored above the pass mark at first attempt. This effect is less marked for PLAB part 1, where the value is only 1% per point scored relative to the pass mark.

We repeated the analyses predicting censure but restricted the dataset to those 1168 PLAB international medical graduates who had been referred at least once in relation to FtP issues within the study exposure period (in contrast to the wider pool of 27,330 PLAB graduates). This was in order to establish which variables predicted eventual censure in this subgroup of referred doctors. Again, the results are depicted in Figs. 1 and 2 (the coefficients are depicted as red squares). As can be seen from the figures the variables that were significantly predictive (*P* < 0.05) of progressing from a referral to censure were male sex (HR, 2.01; 1.41 to 2.88), PLAB part 2 score at first sitting (HR, 0.96; 0.93 to 0.99) and multiple attempts at part 2 (HR, 1.37; 1.01 to 1.85). In contrast, PLAB part 1 scores were more weakly predictive of censure, with those having four or more sittings at the exam having an increased risk of eventual censure following a referral for FtP, compared to those passing first (HR, 1.71; 1.05 to 2.80) or second time (HR, 1.83; 1.05 to 3.20). The full results are depicted in Additional file 1: Table S6. Thus, to summarise, for international medical graduates that were referred in relation to an FtP concern it was mainly male sex and PLAB part 2 performance that predicted who would progress to an actual sanction.

Two variables statistically significantly (at the *P* < 0.05 level) predicted which doctors were eventually censured for purely non-clinical concerns (i.e. professionalism issues only) compared to those who were censured in relation to clinical issues (with or without non-clinical concerns accompanying them). The results are portrayed in Figs. 1 and 2 (the coefficients are depicted as green circles). Firstly, age at registration was associated with being censured purely in relation to professionalism issues (HR, 0.96; 0.93 to 0.99). This suggested that doctors who were older at registration were, on average, less likely to be censured purely in relation to non-clinical concerns. Taken another way, this implied that older doctors had a higher risk of there being some clinical issues raised with their practice as a component of the concern that led to the censure, whether or not wider matters of professionalism were involved. Secondly, IELTS reading scores were associated with the risk of being censured in relation to purely non-clinical issues (HR, 1.33; 1.08 to 1.63). This implies that, for every band extra scored in this IELTS subtest, the risk of being censured for purely professionalism concerns versus those that have some clinical component increases by around 33%. Again, taken another way, this suggests that lower IELTS reading scores are a risk factor for being censured in relation to some issues related to clinical practise, rather than purely non-clinical concerns. In this sense, reading ability may be related in some way to clinical competence. It should be noted that the analysis was limited by the small number of doctors in each category, with only 12 doctors having been censured in relation to ‘purely clinical concerns’, 31 for ‘mixed concerns’ and 172 in relation to ‘purely non-clinical’ issues. The full results are shown in Additional file 1: Table S7.

The results from the ‘phtest’ indicate that the assumption of proportional hazards held for most of the univariable survival analyses performed. There is some evidence that the assumption may have been violated in only six out of the 66 univariable analyses. These analyses involved either male sex or aspects of PLAB part 2 performance.

### Multivariable results

These analyses were aimed at developing models for predicting the risk of a referral and censure in relation to concerns relating to FtP issues in PLAB international medical graduates. Building multivariable models in this case was challenging, as many of the educational predictors would be expected to correlate with each other, causing issues with multicollinearity. For this reason, two sets of models were built; one set that treated the PLAB scores as continuous and one that used the number of PLAB sittings as a predictor.

When predicting referral in relation to FtP as an outcome where PLAB scores were treated as continuous only male sex, higher IELTS speaking scores and the scores for both parts of the PLAB tests at first attempt were independent and significant predictors of the risk of referral. In the second model, where the number of sittings at the PLAB test were entered into the model, the independent and significant predictors of a risk for an FtP referral were male sex, lower IELTS reading score, higher IELTS speaking score and more than one attempt at the PLAB part 1. The full results are contained in Additional file 1: Tables S8 and S9.

Additional analyses were also conducted, aimed at developing models for predicting eventual censure (versus no censure, no referral or referral not ending in censure) for FtP issues in the cohort of PLAB international medical graduates.

In the first model, where the PLAB performance is treated as a continuous variable, male sex (HR, 2.64; 1.83 to 3.80), IELTS speaking score (HR, 1.49; 1.20 to 1.84) and PLAB part 2 score at first attempt (HR, 0.94; 0.91 to 0.97) are all independent and statistically significant predictors of eventual censure (Table 3). Likewise, in the second model, male sex and IELTS speaking scores are also independent and statistically significant (*P* < 0.01) predictors of censure (Table 4). However, from the results in Table 4 it can be seen that those taking four or more attempts at PLAB part 1 are at an increased risk of censure compared to those taking the exam only once (HR, 2.13; 1.26 to 3.59) even after controlling for the potential effects of the other variables in the model. It can also be seen that those that take the PLAB part 2 three or more times are more likely to receive eventual censure than those taking the exam either once (HR, 2.45; 1.44 to 4.18) or twice (HR, 1.90; 1.06 to 3.41).

**Table 3:** Results from a multivariable survival analysis predicting the risk of eventual censure (versus no censure or referral) for Fitness to Practise issues in Professional and Linguistic Assessments Board (PLAB) international medical graduates; in this model, PLAB performance was entered as score (relative to pass mark) at first sitting

Predictor	Hazard ratio	*P*	Lower 95% confidence interval	Upper 95% confidence interval
Male sex	2.64	<0.001	1.83	3.80
Age at registration	1.01	0.41	0.98	1.04
IELTS speaking score	1.49	<0.001	1.20	1.84
PLAB part 1 score at first attempt	0.99	0.12	0.99	1.00
PLAB part 2 score at first attempt	0.94	<0.001	0.91	0.97

**Table 4:** Results from a multivariable survival analysis predicting the risk of eventual censure (versus no censure or referral) for Fitness to Practise issues in Professional and Linguistic Assessments Board (PLAB) international medical graduates; in this model, PLAB performance was entered as number of attempts at each part

Predictor	Hazard ratio	*P*	Lower 95% confidence interval	Upper 95% confidence interval
Male sex	2.73	<0.001	1.90	3.93
Age at registration	1.01	0.54	0.98	1.04
IELTS speaking score	1.39	<0.001	1.13	1.72
Resits – PLAB part 1				
Passing PLAB part 1 at 2nd vs. 1st attempt	1.24	0.25	0.86	1.79
Passing PLAB part 1 at 3rd vs. 1st attempt	1.58	0.08	0.94	2.64
Passing PLAB part 1 at ≥ 4th vs. 1st attempt	2.13	<0.001	1.26	3.59
Passing PLAB part 1 at 3rd vs. 2nd attempt	1.27	0.41	0.72	2.26
Passing PLAB part 1 at ≥ 4th vs. 2nd attempt	1.72	0.07	0.96	3.06
Passing PLAB part 1 at ≥ 4th vs. 3rd attempt	1.35	0.38	0.69	2.64
Resits – PLAB part 2				
Passing PLAB part 2 at 2nd vs. 1st attempt	1.29	0.16	0.90	1.83
Passing PLAB part 2 at ≥ 3rd vs. 1st attempt	2.45	0.001	1.44	4.18
Passing PLAB part 2 at ≥ 3rd vs. 2nd attempt	1.90	0.03	1.06	3.41

Similarly to the univariable results, the phtest suggested that there is no evidence to reject the proportional hazards assumption for the majority of predictors in the models. However, the proportional hazard assumption was rejected according to the phtest for male sex when predicting both time to referral and censure (both *P* < 0.01).

In order to understand the practical implications from these survival analysis results, we ‘simulated’ the effect of limiting the number of sittings for the two parts of the PLAB, enabling a retrospective estimate of the number of international medical graduates that would be excluded from registration on the basis of such a policy. In our previous study of selection into medical school, we introduced the concept of ‘number needed to reject’ (NNR). This value expresses the ratio of acceptable candidates that would need to be excluded by a selection process in order to avoid appointing one candidate likely to have an undesirable outcome (however defined) [30]. This is analogous to the concept of ‘number needed to treat’ in medicine and, at least crudely, represents the effectiveness of a selection method in a specific context. The results of this simulation are shown in Table 5. In this situation, there is a complicating factor in that risk of censure is related to exposure time. In this case, we mitigated this potential confounding effect by only including doctors who had been observed for at least 5 years. In addition, we only included censures that occurred within a 5-year observation period, starting at the point the doctor entered the study. This meant we included data from 21,329 doctors in the analysis, which included 176 individuals who received censure during that defined period (2006 to 2011, inclusive). As can be seen, in terms of NNR, the most favourable conditions would be to have unlimited attempts permitted at part 2 of the PLAB but restrict the number of sittings of part 1 of the exam to three (i.e. two resits would be permitted). This would provide a NNR of 48; i.e. 48 doctors who would not go on to be censured during a 5-year period would have to be excluded in order to prevent the registration of one doctor who was censured during that 5-year exposure period. However, as can be seen in Table 5, the absolute number of censured doctors excluded is very small at 14 (i.e. 8% of the censured doctors). In order to exclude larger numbers of censured doctors, the values in Table 5 suggest that the number of attempts at either part 1 or part 2 of the PLAB would have to be restricted to only one or two sittings. At the extreme end we see that registering only candidates who pass both parts of the PLAB at first attempt might be expected to approximately halve the rate of censures in this group of doctors.

**Table 5:** The number of international medical graduates that would be excluded (*n* = 21,329 doctors in total; 176 censured) under a variety of hypothetical restrictions on the number of attempts at the Professional and Linguistic Assessments Board (PLAB) test

Number of attempts at PLAB Part 2 permitted					
No. of allowable attempts at PLAB Part 1	1	2	3	4	∞
1	79: 9479 (120)	61: 5917 (97)	57: 5364 (94)	56: 5274 (94)	56: 5260 (94)
2	60: 6820 (114)	36: 2555 (71)	28: 1875 (67)	27: 1769 (66)	27: 1752 (65)
3	53: 6052 (114)	24: 1531 (64)	15: 799 (53)	14: 683 (49)	14: 666 (48)
4	45: 5770 (128)	13: 1152 (89)	3: 400 (133)	2: 278 (1139)	2: 260 (130)
∞	45: 5599 (124)	12: 914 (76)	1: 144 (144)	0: 18 (NA)	0: 0 (NA)

The values represent the ratio of censured to uncensured doctors excluded. The NNR ratio is in parentheses

## Discussion

Previous work has shown that doctors who qualify outside the UK (including those from the EEA) are more likely to be referred for FtP issues and are also more likely to experience higher impact decisions at each stage of the GMC process; these observations appeared independent of the enquiry-related and doctor-related characteristics [19]. In the present population of PLAB international medical graduates, we observed that most demographic and PLAB performance indicators were predictive of an FtP referral occurring. In particular, the magnitude of the association we observed between male sex and censure was in keeping with the findings of a previous meta-analytic study [20]. These effects seemed independent of the other variables. Previously, it has been shown that performance at both the IELTS English language test and PLAB exams predicts later achievement in postgraduate medical training in international medical graduates [11, 12]. Likewise, we found that aspects of performance on the PLAB test was predictive of both the likelihood of referral and eventual censure. However, when considering English language competence, the picture seems more complex when predicting FtP issues compared to educational performance. Firstly, we noted that language competence (in terms of reading and listening) was associated with the risk of a referral in relation to FtP concerns. However, the univariable association with English language profile (as indexed by the IELTS scores) and censure was only significant when predicting censure versus ‘no referral, no censure or referral only’. Thus, when modelling the progression to censure in referred doctors no statistically significant (i.e. *P* > 0.05) associations on univariable analysis with IELTS scores were observed. Thus, it may be that English language ability may increase the risk of a complaint or concern to the GMC, but not itself be strongly associated with a risk of actual professional misconduct. For those international doctors who are less confident in understanding English it is easy to see how misunderstandings or communication problems could occur with patients or colleagues. These could potentially trigger complaints or reported concerns to the GMC, which are ultimately closed with no further action.

Performance at PLAB part 1 was also a relatively weak predictor of progression from referral to censure; whilst multiple sittings of that part of the test were associated with an increased risk of progression, the actual score at first attempt was not. Rather, it was achievement at part 2 of the PLAB that was more closely associated with the risk of a doctor progressing from referral to censure, along with male sex. This implies that various aspects of performance can increase the risk of being caught up in the rather broad and non-specific ‘trawler-net’ of the FtP referral process but that, once within this process, relatively few of these variables predict progression to censure. The weak association with PLAB part 1 performance suggests that it is not largely lack of semantic medical knowledge that is associated with the risk of eventual censure for those investigated by the GMC. In contrast, as outlined earlier, PLAB part 2 evaluated procedural skills, which will include ratings of interactions with role played patients. Thus, it is likely to be the capturing of these softer skills by PLAB part 2 that explains this association. Similarly, it is male sex and PLAB part 2 performance that are two of the three statistically significant predictors retained the multivariable models built with censure as the outcome.

There is one puzzling and unexpected predictor that was observed in both the univariable models and retained in the multivariable model predicting censure; paradoxically, higher IELTS speaking score appeared to be a risk factor for censure (compared to no censure/referral not leading to censure); this finding is not easy to explain. However, it could be speculated that high verbal ability, in the absence of a high level of other academic ability, may lead some doctors to attempt to ‘talk their way out of trouble’ if concerns regarding their practise are raised. It also possible that other colleagues assumed a greater competence due to good verbal ability and any support that may have been offered was considered unnecessary in such cases. There is also a clue in the correlation matrix for the predictor variables (Additional file 1: Table S1). The speaking score is the only one of the four IELTS subtest results that correlates positively with age at registration (i.e. older doctors tend to score more on this section of the test). Previously, we have described a complex curvilinear relationship between age and Annual Review of Competence Progression (a rating of postgraduate training performance) outcome in doctors [26], with older doctors generally performing less well. Moreover, doctors who are non-UK graduates and aged above 50 years are over represented in those practitioners who are sanctioned or issued with warnings by the GMC [31]. Thus, it may be that the association with censure and higher levels of spoken English are partly mediated by the age of the doctor. It might also be hypothesised that spoken language ability may be a marker of the country of qualification, and thus be an artefact. For example, some countries tend to more readily access English language media such as music and cinema. In some countries for preference and, perhaps, practical reasons, movies may have subtitles rather than be overdubbed with native actors’ voices. Access to such media may improve spoken language though not necessarily reading, writing and listening. Indeed, there is some debate about whether subtitles in a native language help or hinder language acquisition [32]. In addition, some countries may teach medicine using English. It may be that such countries have cultural practices and expectations, and possibly variation in the nature of their medical training, that differs from those in the West, increasing the risk of disciplinary action by regulators. One Canadian study noted that international doctors from several countries had a higher risk of receiving disciplinary action from the regulator compared to those practitioners trained in North America [33]. This issue could be further explored if both the doctor’s nationality and country (rather than world region) of qualification could be made available for analysis.

Our analysis simulating restrictions on the number of permitted attempts at the PLAB suggests that the proposed limit of four attempts at both parts will not, in itself, substantially reduce the rate of sanctions in international doctors practising in the UK. Such reductions may be possible, but only with more stringent changes to the exam system. This assumes that the PLAB test remains the same as during this study period and it should be highlighted that a number of other changes have already been made to the system. These include increasing the length and number of clinical scenarios in part 2 of the PLAB, the inclusion of additional scenarios which evaluate a candidate’s knowledge of ethics and professionalism, and providing more feedback to candidates [6]. Further changes to the test are planned for September 2017 when the number of attempts at both parts will be limited to four. In addition, from this time onwards, part 2 must be passed within 2, rather than 3, years of passing part 1 [6]. The PLAB test may eventually be superseded by a UK national medical licensing exam [34].

There are a number of additional limitations worth noting. Firstly, these findings only apply to international medical graduates who used the PLAB system to demonstrate their clinical skills and knowledge, rather than by other means. Secondly, the data supplied for the research were only available on region, rather than country of origin (in order to protect anonymity), precluding more detailed analysis. Thirdly, an obvious limitation was the small number of doctors in the dataset who were eventually censured. When the categories were divided, even crudely, this led to very sparse outcomes and would have negatively impacted on study power. Fourthly, the dataset will not have captured FtP cases relating to doctors in the sample that were closed prior to 2006. These limitations must be borne in mind when interpreting the findings. Finally, survival analysis controls for the effects of time but assumes that the hazard ratios act at a constant rate (the parallel hazard ratio assumption) across the period of risk. In medicine, this may not be a plausible assumption, given the different career stages and varying responsibility levels which attend these roles. We performed formal testing to check the validity of such an assumption and it was supported in the vast majority of analyses. However, in several cases, there was evidence to suggest the assumption may not hold. For males, this may be due to longer practising male doctors being at a raised risk of referral or sanction in relation to FtP issues. Such doctors may have been relatively over represented in the first part of the observation period, having already been registered in some cases for many years prior to the start of the study (2006). The proportional hazard assumption was not always upheld in analyses involving PLAB part 2 performance. This may have been because some doctors passed the test several years before the start of the study observation period and thus the performance on the exam may have been more relevant for those who had taken the test more recently. As the descriptive statistics show, referral in relation to an FtP concern is, in itself, a poor predictor of eventual censure, although this may also depend on the source of the referral (not included in the present models). Moreover, medical speciality may have an influence on the likelihood of censure following a referral for FtP issues. A previous study of FtP processes categorised doctors as those who working in general practice or in a hospital speciality but did not utilise this factor as a predictor in their analyses [19]. The role of speciality could be investigated in more detail in future research, especially for those where there is a particular emphasis on interpersonal and communication skills, such as psychiatry.

It should also be stressed that, at the time of the study, neither the IELTS nor the PLAB test explicitly evaluated key aspects of professionalism, such as whether a candidate understands the values deemed desirable, and indeed now mandated, in UK health practitioners [35]. This is especially important as the vast majority of FtP concerns communicated to the GMC are not solely related to clinical competence. As outlined earlier, some of the procedural skills (such as inter-personal communication) will have been evaluated as part of the PLAB system and, from September 2016, additional scenarios related to ethics and professionalism have been included in part 2. The GMC itself outlines the expectations for the conduct of registered doctors’ and standards of professionalism in the ‘Good Medical Practice’ guide [36]. However, knowledge of professionalism may be more efficiently measured by other methods; one such approach has been the use of Situational Judgment Tests (SJTs). In an SJT, a series of scenarios that challenge professionalism are presented. The candidate must respond in a way which demonstrates a knowledge of appropriate professional behaviours. This could be, for example, by selecting or ranking the most appropriate behaviour required in that situation, from a list of alternatives [37]. The use of SJTs has already been introduced into various stages of medical selection in the UK and there is a possibility that such an approach will also be implemented as part of the GMC registration process for international doctors [25].

Overall, these results highlight the role that linguistic ability and communication plays in both the risk of referral for FtP issues, as well as eventual censure. Our findings do provide some support for the recently implemented and proposed changes by the GMC; raising the standards of the language requirements for registration as well as ristricting the number of permitted sittings on the PLAB. Limiting the number of attempts at high stakes exams is a contentious issue. In postgraduate medical exams there is some evidence that scores (and hence assumed ability) continue to increase, on average, even after many resits; this has been suggested as a rationale for placing no limits on the number of attempts [38]. However, one would have to show that such increased scores, on repeated sitting, were still an accurate marker of the construct under evaluation, or possibly perform some manner of adjustment for the number of resits. As mentioned in the introduction, the Australian Medical Council currently allows such unlimited attempts at their professional exams. Nevertheless, it may be that regulatory bodies should perhaps require evidence of successful further study with a minimum ‘refractory period’ before allowing candidates to enter upon a second ‘series’ of tests. The length of such a refractory period would be an interesting area for further research. On the other hand, with a greater number of resits, chance will play an increasing role in helping a candidate ultimately pass the test [38]. This factor makes an argument for limiting exam sittings and timings. In this instance, where there is an external criterion (i.e. the risk of eventual censure) one can make recommendations on the number of resits on a test that should be permitted on this basis. Specifically, for the PLAB exam, we suggest that limiting the number of attempts at part 1 to three sittings may be the optimal restriction in these circumstances. Our findings suggest this is the least stringent limit that would be required if a strengthened system was to lead to appreciably reduced rates of sanction in international medical graduates working in the UK. Thus, we would recommend imposing this further restriction on the number of times the parts of the PLAB could be taken, beyond the four sittings for each part proposed. It should be noted there is some paradox here in that it was performance at part 2 of the PLAB that predicted progression from referral to censure more strongly than achievement at part 1 of the test. As PLAB part 2 is an observed practical test, even prior to the recent changes, there would have been opportunities for candidates to demonstrate some of the behaviours expected by the GMC when interacting with patients in UK health services culture (or the converse for that matter). However, the vast majority of doctors pass part 2 at either the first or second attempt. Thus, there is little absolute difference in the numbers of candidates who pass part 2 at the fourth rather than the third attempt, making imposing such a limit relatively ineffective. Secondly, in order to receive a sanction, by definition, a doctor must first be referred to the GMC. Thus, it may be that performance at PLAB part 1 may be more of a marker for referral than eventual censure, but that this is a necessary gateway to eventual sanction. As highlighted in Table 2, very few doctors are eventually sanctioned in relation to purely clinical concerns. However, suspected deficiencies in medical knowledge could draw attention to the wider aspects of practice in a doctor, who is eventually censured predominantly or solely in relation to professionalism issues.

## Conclusion

Our results suggest that evaluating language competency and clinical skills and knowledge may be useful in reducing rates of FtP issues in international graduates, albeit mainly via indirect effects. However, ultimately, the focus of medical regulators should also be on assessing whether doctors working outside of their countries of qualification understand, and are likely to exhibit, the professional behaviours appropriate to the health services culture they intend to work in. Thus, we would recommend further enhancing the evaluation of knowledge and behaviour in relation to medical professionalism in a UK context as part of the PLAB test or wider registration process. There are also examples of pilot schemes that aim to support international graduates in enhancing their communication skills and cultural competence [39]. Such steps are likely to help prevent international doctors from being exposed to stressful, but ultimately groundless, complaints and investigations. Importantly, these measures would also reduce the risk that patients are exposed to from the small minority of practitioners that may exhibit impaired professional values and conduct. There are high financial costs and often much suffering associated with professional malpractice in medicine. Thus, even measures that modestly reduce the risk of such situations occurring may be cost-effective.

## Additional file

Additional file 1: Supplementary Results Appendix. (DOCX 46 kb)

## Abbreviations

EEA: European Economic Area
FtP: fitness to practise
GMC: General Medical Council
HR: hazard ratio
IELTS: International English Language Test System
NNR: number needed to reject
PLAB: Professional and Linguistic Assessment Board

## Footnotes

1 Note that each recorded referral or censure can be in relation to multiple categories of concern.

2 In this context, a breach of ‘compliance’ means breaching terms of conditions or undertakings.
