# Supplementary material for: Predicting fitness to practise events in international medical graduates who registered as UK doctors via the Professional and Linguistic Assessments Board (PLAB) system: a national cohort study
Source: BMC Med. 2017 Mar 20;15:66. doi: 10.1186/s12916-017-0829-1 (PMC5357806; doi:10.1186/s12916-017-0829-1)
Supplement: Additional file 1: — Supplementary Results Appendix. (DOCX 46 kb) [file 12916_2017_829_MOESM1_ESM.docx]

Supplementary Results Appendix to:

**Predicting fitness to practise events in international medical graduates who registered as UK doctors via the Professional and Linguistic Assessments Board (PLAB) system: a national cohort study**

Paul A Tiffin MD, Lewis W Paton PhD, Lazaro M Mwandigha MSc, John C McLachlan PhD, & Jan Illing PhD

This supplementary appendix contains the full tabulated results from the survival analyses related to the survival analyses in the paper. Specifically the full results from the survival analyses predicting referral for fitness to practise (FtP) issues are presented, along with the tabulated results from those with actual censure as an outcome (presented in graphical form in the main paper in Figs. 1 and 2).

**A: Correlations between continuous predictor variables**

As part of the results section in the main paper we allude to the degree of correlation between the main continuous predictor variables. These are shown in Table S1 below. All values are statistically significant at the p<0.0001 level except for the correlation between PLAB part 1 score at first sitting and the IELTS speaking score (r=0.01, p=0.03). In the main paper we also highlight that only the correlation between age and the ‘speaking’ subtest score of the IELTS is positive in direction. Thus, this is the only part of the English language test that, on average, appears to improve with the doctor’s increasing age.

|  | Age at registration (years) | IELTS reading | IELTS writing | IELTS comp. | IELTS speaking | PLAB part 1 score | PLAB part 2 score |
| --- | --- | --- | --- | --- | --- | --- | --- |
| Age at registration (years) |  |  |  |  |  |  |  |
| IELTS reading | -0.18 |  |  |  |  |  |  |
| IELTS writing | -0.03 | 0.18 |  |  |  |  |  |
| IELTS listening | -0.15 | 0.40 | 0.15 |  |  |  |  |
| IELTS speaking | 0.06 | 0.18 | 0.23 | 0.2 |  |  |  |
| PLAB part 1 score | -0.15 | 0.24 | 0.11 | 0.15 | 0.01 |  |  |
| PLAB part 2 score | -0.14 | 0.21 | 0.16 | 0.21 | 0.22 | 0.27 |  |

Table S1. Matrix of correlations between the continuous predictor variables.

**B: Results from univariable survival analysis: Referral for FtP concerns**

| **Predictor** | **Hazard ratio** | **P** | **Lower 95% Confidence Interval** | **Upper 95% Confidence Interval** |
| --- | --- | --- | --- | --- |
| Male sex | 1.52 | <0.001 | 1.34 | 1.72 |
| Age at registration | 1.02 | 0.001 | 1.01 | 1.03 |
| IELTS overall score | 0.94 | 0.33 | 0.82 | 1.07 |
| IELTS reading score | 0.87 | <0.001 | 0.80 | 0.94 |
| IELTS speaking score | 1.20 | <0.001 | 1.10 | 1.31 |
| IELTS listening score | 0.89 | 0.01 | 0.82 | 0.96 |
| IELTS writing score | 1.00 | 0.89 | 0.94 | 1.07 |
| PLAB part 1 score at first attempt | 0.99 | <0.001 | 0.99 | 0.99 |
| PLAB part 2 score at first attempt | 0.97 | <0.001 | 0.96 | 0.99 |
| Multiple attempts at PLAB part 1 | 1.38 | <0.001 | 1.23 | 1.56 |
| Multiple attempts at PLAB part 2 | 1.17 | 0.02 | 1.02 | 1.35 |
| **Resits- PLAB part 1** | | | | |
| Passing PLAB part 1 at 2^nd^ vs 1^st^ attempt | 1.29 | <0.001 | 1.12 | 1.50 |
| Passing PLAB part 1 at 3^rd^ vs 1^st^ attempt | 1.42 | <0.001 | 1.13 | 1.77 |
| Passing PLAB part 1 at ≥4^th^ vs 1^st^ attempt | 1.74 | <0.001 | 1.37 | 2.22 |
| Passing PLAB part 1 at 3^rd^ vs 2^nd^ attempt | 1.10 | 0.47 | 0.87 | 1.40 |
| Passing PLAB part 1 at ≥4^th^ vs 2^nd^ attempt | 1.35 | 0.03 | 1.03 | 1.75 |
| Passing PLAB part 1 at ≥4^th^ vs 3^rd^ attempt | 1.23 | 0.20 | 0.90 | 1.68 |
| **Resits- PLAB part 2** | | | | |
| Passing PLAB part 2 at 2^nd^ vs 1^st^ attempt | 1.13 | 0.10 | 0.98 | 1.32 |
| Passing PLAB part 2 at ≥3^rd^ vs 1^st^ attempt | 1.41 | 0.03 | 1.05 | 1.90 |
| Passing PLAB part 2 at 3^rd^ vs 2^nd^ attempt | 1.24 | 0.18 | 0.90 | 1.71 |

Table S2. Results from univariable survival analyses predicting the relative risk of a referral for any fitness to practise issue in PLAB international medical graduates (N=27,330).

We observed that in this sample of international medical graduates who demonstrated their clinical knowledge and skills via the PLAB, most demographic and PLAB performance indicators were predictive of an FtP referral occurring (Table S2). The only exceptions to this were the overall IELTS score as well as the IELTS *writing* score. Men were 37% more likely than women to experience a

referral for any FtP issue when practising. Increasing age at registration appears a slight, but significant, risk for FtP referral. Higher IELTS *reading* and *listening* scores are protective of FtP, while a higher *speaking* score appears a modest, though statistically significant, risk factor for FtP referral. Multiple attempts at PLAB part 1 or PLAB part 2 are a generally a risk factor for FtP referral.

| **Predictor** | **Hazard Ratio** | **P** | **Lower 95% Confidence Interval** | **Upper 95% Confidence Interval** |
| --- | --- | --- | --- | --- |
| Male sex | 1.26 | 0.13 | 0.93 | 1.71 |
| Age at registration | 1.04 | 0.001 | 1.02 | 1.07 |
| IELTS overall score | 1.03 | 0.84 | 0.75 | 1.42 |
| IELTS reading score | 0.88 | 0.20 | 0.73 | 1.07 |
| IELTS speaking score | 1.12 | 0.35 | 0.89 | 1.40 |
| IELTS listening score | 0.98 | 0.87 | 0.80 | 1.21 |
| IELTS writing score | 1.03 | 0.59 | 0.93 | 1.13 |
| PLAB part 1 score at first attempt | 1.00 | 0.98 | 0.99 | 1.01 |
| PLAB part 2 score at first attempt | 0.99 | 0.42 | 0.96 | 1.02 |
| Multiple attempts at PLAB part 1 | 0.99 | 0.96 | 0.72 | 1.36 |
| Multiple attempts at PLAB part 2 | 0.85 | 0.39 | 0.58 | 1.23 |
| **Resits- PLAB part 1** | | | | |
| Passing PLAB part 1 at 2^nd^ vs 1^st^ attempt | 0.96 | 0.84 | 0.66 | 1.41 |
| Passing PLAB part 1 at 3^rd^ vs 1^st^ attempt | 1.01 | 0.97 | 0.55 | 1.87 |
| Passing PLAB part 1 at ≥4^th^ vs 1^st^ attempt | 1.10 | 0.80 | 0.54 | 2.24 |
| Passing PLAB part 1 at 3^rd^ vs 2^nd^ attempt | 1.05 | 0.89 | 0.53 | 2.09 |
| Passing PLAB part 1 at ≥4^th^ vs 2^nd^ attempt | 1.14 | 0.74 | 0.53 | 2.48 |
| Passing PLAB part 1 at ≥4^th^ vs 3^rd^ attempt | 1.09 | 0.86 | 0.44 | 2.70 |
| **Resits- PLAB part 2** | | | | |
| Passing PLAB part 2 at 2^nd^ vs 1^st^ attempt | 0.84 | 0.40 | 0.56 | 1.26 |
| Passing PLAB part 2 at ≥3^rd^ vs 1^st^ attempt | 0.89 | 0.80 | 0.37 | 2.17 |
| Passing PLAB part 2 at 3^rd^ vs 2^nd^ attempt | 1.06 | 0.91 | 0.41 | 2.74 |

Table S3. Results from univariable survival analyses predicting the relative risk of a referral for a fitness to practise issue related to *purely clinical concerns* in a PLAB international medical graduates (versus, other category of referral or no referral at all, N=27,330).

In contrast to FtP referrals in general, male sex was not a significant risk factor for risk of being referred in relation to purely clinical concerns (as opposed to no referrals, or referrals that involved *non-clinical concerns*). The results are shown in Table S3. However, this finding may have partly been due to a lack of study power (i.e. a type II error) because of the relatively smaller numbers involved.

| **Predictor** | **Hazard Ratio** | **P** | **Lower 95% Confidence Interval** | **Upper 95% Confidence Interval** |
| --- | --- | --- | --- | --- |
| Male gender | 0.81 | 0.19 | 0.60 | 1.10 |
| Age at registration | 1.02 | 0.06 | 1.00 | 1.05 |
| IELTS overall score | 1.10 | 0.53 | 0.81 | 1.50 |
| IELTS reading score | 1.01 | 0.90 | 0.84 | 1.22 |
| IELTS speaking score | 0.95 | 0.63 | 0.75 | 1.18 |
| IELTS listening score | 1.12 | 0.25 | 0.92 | 1.38 |
| IELTS writing score | 1.04 | 0.72 | 0.84 | 1.28 |
| PLAB part 1 score at first attempt | 1.01 | 0.02 | 1.00 | 1.02 |
| PLAB part 2 score at first attempt | 1.01 | 0.52 | 0.98 | 1.04 |
| Multiple attempts at PLAB part 1 | 0.72 | 0.04 | 0.52 | 0.99 |
| Multiple attempts at PLAB part 2 | 0.72 | 0.08 | 0.49 | 1.04 |
| **Resits- PLAB part 1** | | | | |
| Passing PLAB part 1 at 2^nd^ vs 1^st^ attempt | 0.73 | 0.11 | 0.50 | 1.08 |
| Passing PLAB part 1 at 3^rd^ vs 1^st^ attempt | 0.71 | 0.26 | 0.38 | 1.30 |
| Passing PLAB part 1 at ≥4^th^ vs 1^st^ attempt | 0.67 | 0.28 | 0.33 | 1.37 |
| Passing PLAB part 1 at 3^rd^ vs 2^nd^ attempt | 0.96 | 0.91 | 0.48 | 1.90 |
| Passing PLAB part 1 at ≥4^th^ vs 2^nd^ attempt | 0.92 | 0.82 | 0.42 | 1.99 |
| Passing PLAB part 1 at ≥4^th^ vs 3^rd^ attempt | 0.95 | 0.92 | 0.38 | 2.37 |
| **Resits- PLAB part 2** | | | | |
| Passing PLAB part 2 at 2^nd^ vs 1^st^ attempt | 0.74 | 0.15 | 0.50 | 1.11 |
| Passing PLAB part 2 at ≥3^rd^ vs 1^st^ attempt | 0.59 | 0.25 | 0.24 | 1.45 |
| Passing PLAB part 2 at 3^rd^ vs 2^nd^ attempt | 0.80 | 0.64 | 0.31 | 2.07 |

Table S4. Results from univariable survival analyses predicting the relative risk of a referral for a fitness to practise issue purely related to *clinical* concerns in a PLAB international medical graduates, compared to a referral in relation to *non-clinical* concerns (with or without clinical issues raised, N=1,168).

Indeed, only 196 of the doctors were referred purely on clinical grounds. Only age at registration was a significant predictor of FtP referral in this group - in general older doctors at registration were at higher risk of being referred purely in relation to clinical concerns.

Previous work has suggested a complex curvilinear relationship between age at Annual Review of Competence Progression (ARCP) and outcomes in doctors [1] but this finding is in keeping with the proposition that older doctors may be more prone to concerns relating to their clinical skills- perhaps due to a perception of over confidence with experience, or an impression that they are not keeping up to date with innovations and medical advances in practice.

We also explored the predictors for being referred for *purely clinical concerns*, compared to a referral in relation to *non-clinical* concerns (with or without clinical issues raised). These analyses were conducted as it was hypothesised that, as the PLAB is a clinical exam system, performance may especially predict FtP issues in relation to purely clinical work. The results are depicted in Table S4. Again, a doctor’s age at registration was a predictor of borderline statistical significance (p=0.06) for a referral for a purely clinical FtP concern. Paradoxically, a better PLAB part 1 score at first attempt was also a risk factor for being referred for *purely clinical concerns* (vs *mixed* or *non-clinical* issues), and multiple attempts at the exam a (borderline) protective factor. This may be an artefact of more able candidates working in specialities (such as surgery) that may be more prone to complaints based on purely clinical issues. It should also be noted that almost no FtP referrals purely based on clinical concerns results in eventual censure. Therefore referral in this category is unlikely to be a reliable marker of clinical competence in an international medical graduate.

**C: Results from univariable survival analysis: censure for FtP concerns**

Tables S5-S7 relate to FtP concerns which result in a censure (i.e. sanction of some kind). They correspond to the results shown in Figs. 1 and 2 in the main text.

| **Predictor** | **Hazard Ratio** | **P** | **Lower 95% Confidence Interval** | **Upper 95% Confidence Interval** |
| --- | --- | --- | --- | --- |
|  |  |  |  |  |
| Male sex | 2.88 | <0.001 | 2.01 | 4.13 |
| Age at registration | 1.02 | 0.12 | 0.99 | 1.05 |
| IELTS overall score | 0.80 | 0.16 | 0.58 | 1.09 |
| IELTS reading score | 0.79 | 0.01 | 0.65 | 0.94 |
| IELTS speaking score | 1.28 | 0.02 | 1.04 | 1.57 |
| IELTS listening score | 0.76 | 0.01 | 0.62 | 0.93 |
| IELTS writing score | 0.94 | 0.54 | 0.77 | 1.15 |
| PLAB part 1 score at first attempt | 0.99 | 0.01 | 0.98 | 1.00 |
| PLAB part 2 score at first attempt | 0.94 | <0.001 | 0.91 | 0.97 |
| Multiple attempts at PLAB part 1 | 1.49 | 0.01 | 1.12 | 1.97 |
| Multiple attempts at PLAB part 2 | 1.57 | 0.003 | 1.16 | 2.13 |
| **Resits- PLAB part 1** | | | | |
| Passing PLAB part 1 at 2^nd^ vs 1^st^ attempt | 1.22 | 0.28 | 0.85 | 1.74 |
| Passing PLAB part 1 at 3^rd^ vs 1^st^ attempt | 1.61 | 0.07 | 0.97 | 2.66 |
| Passing PLAB part 1 at ≥4^th^ vs 1^st^ attempt | 2.51 | <0.001 | 1.54 | 4.11 |
| Passing PLAB part 1 at 3^rd^ vs 2^nd^ attempt | 1.32 | 0.34 | 0.75 | 2.34 |
| Passing PLAB part 1 at ≥4^th^ vs 2^nd^ attempt | 2.07 | 0.01 | 1.18 | 3.61 |
| Passing PLAB part 1 at ≥4^th^ vs 3^rd^ attempt | 1.56 | 0.19 | 0.81 | 3.03 |
| **Resits- PLAB part 2** | | | | |
| Passing PLAB part 2 at 2^nd^ vs 1^st^ attempt | 1.34 | 0.10 | 0.95 | 1.88 |
| Passing PLAB part 2 at ≥3^rd^ vs 1^st^ attempt | 2.95 | <0.001 | 1.76 | 4.94 |
| Passing PLAB part 2 at 3^rd^ vs 2^nd^ attempt | 2.21 | 0.01 | 1.24 | 3.93 |

Table S5. Results from univariable survival analyses predicting the relative risk of an eventual censure for fitness to practise issues (compared to no referral or referrals that do not result in censure) in PLAB international medical graduates (N=27,330).

| **Predictor** | **Hazard Ratio** | **P** | **Lower 95% Confidence Interval** | **Upper 95% Confidence Interval** |
| --- | --- | --- | --- | --- |
| Male gender | 2.01 | <0.001 | 1.41 | 2.88 |
| Age at registration | 1.00 | 0.86 | 0.98 | 1.03 |
| IELTS overall score | 0.85 | 0.29 | 0.62 | 1.15 |
| IELTS reading score | 0.90 | 0.26 | 0.76 | 1.08 |
| IELTS speaking score | 1.10 | 0.38 | 0.89 | 1.35 |
| IELTS listening score | 0.86 | 0.13 | 0.70 | 1.04 |
| IELTS writing score | 0.89 | 0.26 | 0.73 | 1.09 |
| PLAB part 1 score at first attempt | 1.00 | 0.78 | 0.99 | 1.01 |
| PLAB part 2 score at first attempt | 0.96 | 0.01 | 0.93 | 0.99 |
| Multiple attempts at PLAB part 1 | 1.11 | 0.48 | 0.83 | 1.47 |
| Multiple attempts at PLAB part 2 | 1.37 | 0.04 | 1.01 | 1.85 |
| **Resits- PLAB part 1** |  |  |  |  |
| Passing PLAB part 1 at 2^nd^ vs 1^st^ attempt | 0.94 | 0.72 | 0.66 | 1.34 |
| Passing PLAB part 1 at 3^rd^ vs 1^st^ attempt | 1.16 | 0.56 | 0.70 | 1.92 |
| Passing PLAB part 1 at ≥4^th^ vs 1^st^ attempt | 1.71 | 0.03 | 1.05 | 2.80 |
| Passing PLAB part 1 at 3^rd^ vs 2^nd^ attempt | 1.24 | 0.46 | 0.70 | 2.19 |
| Passing PLAB part 1 at ≥4^th^ vs 2^nd^ attempt | 1.83 | 0.03 | 1.05 | 3.20 |
| Passing PLAB part 1 at ≥4^th^ vs 3^rd^ attempt | 1.47 | 0.25 | 0.76 | 2.86 |
| **Resits- PLAB part 2** |  |  |  |  |
| Passing PLAB part 2 at 2^nd^ vs 1^st^ attempt | 1.20 | 0.29 | 0.86 | 1.69 |
| Passing PLAB part 2 at ≥3^rd^ vs 1^st^ attempt | 2.15 | <0.01 | 1.28 | 3.60 |
| Passing PLAB part 2 at 3^rd^ vs 2^nd^ attempt | 1.79 | 0.05 | 1.00 | 3.18 |
|  |  |  |  |  |

Table S6. Results from univariable survival analyses predicting the relative risk of an eventual censure in PLAB international medical graduates referred to the GMC in relation to fitness to practise concerns (N=1,168).

| **Predictor** | **Hazard Ratio** | **P** | **Lower 95% Confidence Interval** | **Upper 95% Confidence Interval** |
| --- | --- | --- | --- | --- |
| Male gender | 1.45 | 0.08 | 0.96 | 2.18 |
| Age at registration | 0.96 | 0.01 | 0.93 | 0.99 |
| IELTS overall score | 1.33 | 0.15 | 0.90 | 1.96 |
| IELTS reading score | 1.33 | 0.01 | 1.08 | 1.63 |
| IELTS speaking score | 0.85 | 0.18 | 0.66 | 1.08 |
| IELTS listening score | 1.12 | 0.30 | 0.91 | 1.37 |
| IELTS writing score | 1.04 | 0.74 | 0.83 | 1.31 |
| PLAB part 1 score at first attempt | 1.00 | 0.66 | 0.99 | 1.01 |
| PLAB part 2 score at first attempt | 1.03 | 0.07 | 1.00 | 1.07 |
| Multiple attempts at PLAB part 1 | 0.92 | 0.60 | 0.67 | 1.26 |
| Multiple attempts at PLAB part 2 | 0.83 | 0.29 | 0.59 | 1.17 |
| **Resits- PLAB part 1** | | | | |
| Passing PLAB part 1 at 2^nd^ vs 1^st^ attempt | 1.10 | 0.63 | 0.75 | 1.62 |
| Passing PLAB part 1 at 3^rd^ vs 1^st^ attempt | 0.77 | 0.38 | 0.43 | 1.37 |
| Passing PLAB part 1 at ≥4^th^ vs 1^st^ attempt | 0.71 | 0.29 | 0.38 | 1.33 |
| Passing PLAB part 1 at 3^rd^ vs 2^nd^ attempt | 0.70 | 0.27 | 0.37 | 1.33 |
| Passing PLAB part 1 at ≥4^th^ vs 2^nd^ attempt | 0.65 | 0.21 | 0.33 | 1.28 |
| Passing PLAB part 1 at ≥4^th^ vs 3^rd^ attempt | 0.93 | 0.85 | 0.41 | 2.07 |
| **Resits- PLAB part 2** | | | | |
| Passing PLAB part 2 at 2^nd^ vs 1^st^ attempt | 0.90 | 0.57 | 0.61 | 1.31 |
| Passing PLAB part 2 at ≥3^rd^ vs 1^st^ attempt | 0.69 | 0.22 | 0.38 | 1.25 |
| Passing PLAB part 2 at ≥3^rd^ vs 2^nd^ attempt | 0.77 | 0.44 | 0.40 | 1.49 |

Table S7. Results from univariable survival analyses predicting the relative risk of an eventual censure for fitness to practise issues in relation to *purely non-clinical concerns* (in contrast to censure involving *clinical issues*, with or without *non-clinical concerns,* N=210).

**D: Results from multivariable survival analysis: Referral for FtP concerns**

Table S8 depicts the results for any type of FtP referral, when PLAB score was entered as score (relative to the pass mark) at first sitting of the test. Only male sex, IELTS speaking score, and the scores for both parts of the PLAB tests were independent significant predictors of the risk of referral.

| **Predictor** | **Hazard Ratio** | **P** | **Lower 95% Confidence Interval** | **Upper 95% Confidence Interval** |
| --- | --- | --- | --- | --- |
| Male sex | 1.55 | <0.001 | 1.35 | 1.77 |
| Age at registration | 1.01 | 0.35 | 0.99 | 1.02 |
| IELTS reading score | 0.92 | 0.07 | 0.85 | 1.01 |
| IELTS speaking score | 1.31 | <0.001 | 1.19 | 1.44 |
| IELTS listening score | 0.95 | 0.30 | 0.87 | 1.04 |
| PLAB part 1 score at first attempt | 0.99 | <0.001 | 0.99 | 1.00 |
| PLAB part 2 score at first attempt | 0.98 | 0.02 | 0.97 | 1.00 |

Table S8. Results from a multivariable survival analysis predicting the risk of a referral in relation to Fitness to Practise concerns in PLAB international medical graduates. In this model PLAB performance was entered as score (relative to pass mark) at first sitting (N=25,764, i.e. those with complete data on all predictors).

Table S9 depicts the results when the number of sittings of the PLAB tests was entered into the model. As can be seen the independent and statistically significant (p<0.05) predictors of a risk of referral were male sex, age at registration, IELTS speaking score, more than one attempt at PLAB part 1, and more than one attempt at PLAB part 2.

| **Predictor** | **Hazard Ratio** | **P** | **Lower 95% Confidence Interval** | **Upper 95% Confidence Interval** |
| --- | --- | --- | --- | --- |
| Male sex | 1.57 | <0.001 | 1.37 | 1.79 |
| Age at registration | 1.01 | 0.37 | 0.99 | 1.02 |
| IELTS reading score | 0.91 | 0.03 | 0.83 | 0.99 |
| IELTS speaking score | 1.29 | <0.001 | 1.17 | 1.42 |
| IELTS listening score | 0.94 | 0.17 | 0.86 | 1.03 |
| **Resits- PLAB part 1** | | | | |
| Passing PLAB part 1 at 2^nd^ vs 1^st^ attempt | 1.27 | <0.01 | 1.09 | 1.48 |
| Passing PLAB part 1 at 3^rd^ vs 1^st^ attempt | 1.31 | <0.01 | 1.03 | 1.66 |
| Passing PLAB part 1 at ≥4^th^ vs 1^st^ attempt | 1.62 | <0.001 | 1.26 | 2.08 |
| Passing PLAB part 1 at 3^rd^ vs 2^nd^ attempt | 1.03 | 0.82 | 0.80 | 1.33 |
| Passing PLAB part 1 at ≥4^th^ vs 2^nd^ attempt | 1.27 | 0.08 | 0.97 | 1.67 |
| Passing PLAB part 1 at ≥4^th^ vs 3^rd^ attempt | 1.24 | 0.20 | 0.90 | 1.71 |
| **Resits- PLAB part 2** | | | | |
| Passing PLAB part 2 at 2^nd^ vs 1^st^ attempt | 1.10 | 0.25 | 0.94 | 1.28 |
| Passing PLAB part 2 at ≥3^rd^ vs 1^st^ attempt | 1.19 | 0.27 | 0.87 | 1.62 |
| Passing PLAB part 2 at ≥3^rd^ vs 2^nd^ attempt | 1.09 | 0.63 | 0.78 | 1.51 |

Table S9. Results from a multivariable survival analysis predicting the risk of referral in relation to Fitness to Practise concerns in PLAB international medical graduates. In this model PLAB performance was entered as number of attempts at each part sitting (N=25,764, i.e. those with complete data on all predictors).

**Reference**

Tiffin PA, Illing J, Webster LAD, McLachlan JC: **The validity of the Professional and Linguistic Assessments Board (PLAB) exam: Research report.** London: The GMC; 2013.
